# Supplementary material for: Experimental and theoretical model of microvascular network remodeling and blood flow redistribution following minimally invasive microvessel laser ablation
Source: Sci Rep. 2024 Apr 16;14:8767. doi: 10.1038/s41598-024-59296-w (PMC11021487; doi:10.1038/s41598-024-59296-w)
Supplement: Supplementary file 1 — Supplementary Information 1. [file 41598_2024_59296_MOESM1_ESM.pdf]

Supplementary Figure 1.

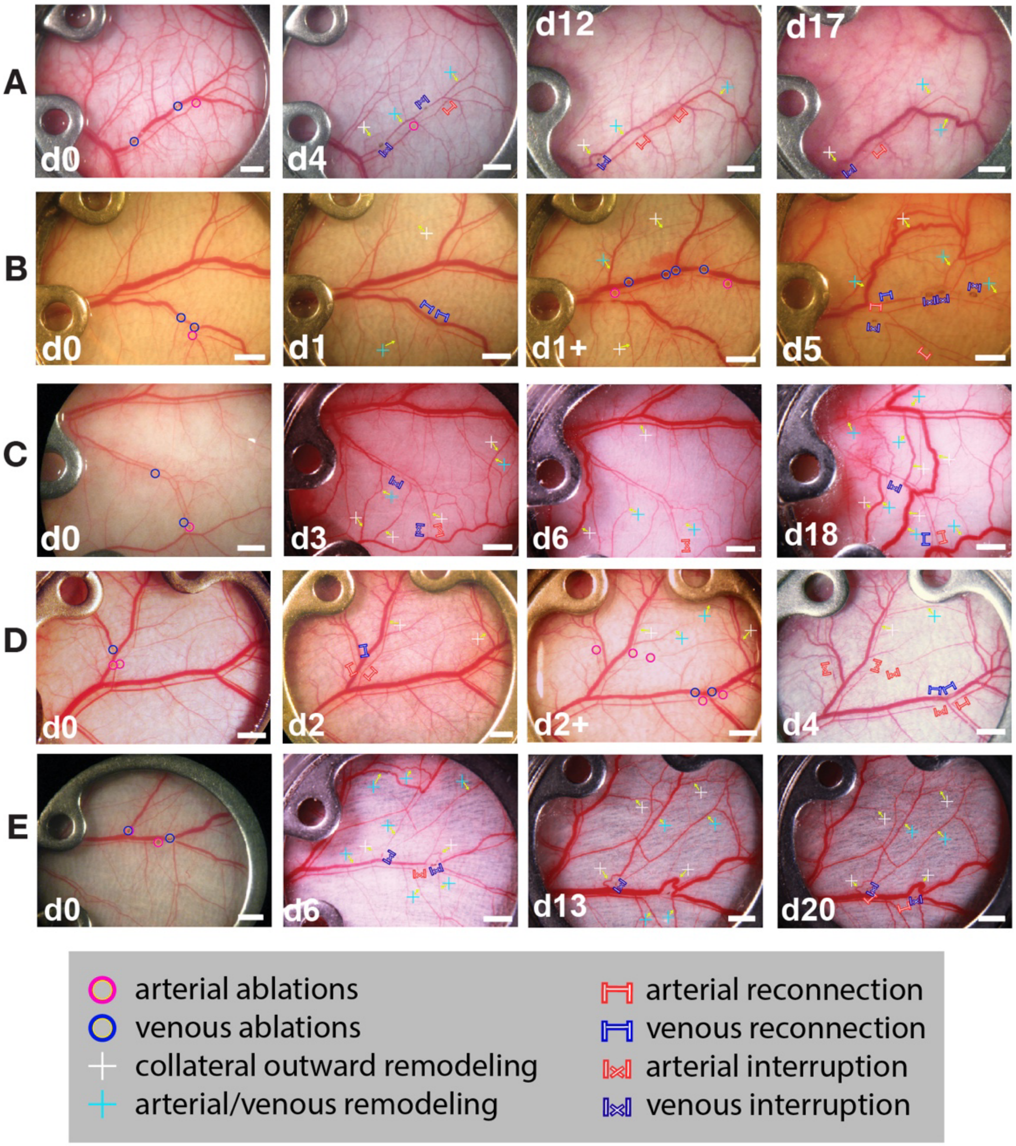

Supplementary Figure 1. The time course and patterns of microvascular network remodeling following laser ablation in five DSFC experiments. Each row (A-E) corresponds to a distinct animal experiment, with Mice A-D showcasing various remodeling patterns. Additionally, we extensively analyze Mouse E, chosen as a representative case, to provide comprehensive anatomical data for the biological and mathematical modeling study. Circles denote the locations of laser ablations: pink for arterial and blue for venous ablations. White “+” sign

indicates collateral outward remodeling, blue “+” sign represent arterial/venous branch segment outward or inward remodeling. The “-“ and “x” marks between bars represent reconnection and interruption for arterial (red) and venous segments. All specimens exhibit significant remodeling at all time points, as detailed in sTables 2-5 and summarized in sTable 6. In Mouse A, proximal venous ablation was bypassed through an existing transverse venule, which remodeled outward to match the initial vein diameter. The distal venous ablation revascularized by day 12, while the main vein initially remodeled inward until day 12 and returned to its pre-ablation diameter by day 17. Arterial ablations and one venous ablation reopened by day 12 in Row A. In Mouse B, venous ablations were either bypassed by outward remodeling of transverse veins (Mouse B, d5, upper half) or caused inward remodeling of the main venous branch (Mouse B, d5, lower half). Mouse C features venous ablations bypassed by pronounced collateral development, while the arterial ablation revascularized. In Mouse D, initial arterial and venous ablations reopened at day 2. Other ablations were performed on the main artery and vein and two of their branches to induce more permanent flow changes. In Mouse E, all remodeling patterns are evident, albeit at varying time courses. Venous ablations revascularize through collateral growth, while arterial occlusions reopen. Most vessels exhibit visible remodeling, with diameter data further described and modeled in the subsequent sections of the study.

Supplementary Figure 2.

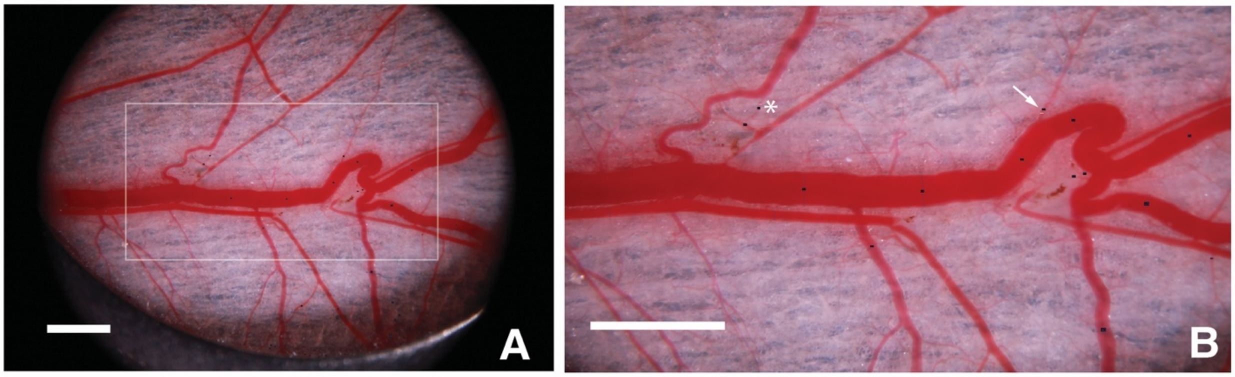

Supplementary Figure 2. Vessel diameter measurement procedure. Diameter were measure from high magnification bright field images (A) using ImageJ. B: A higher magnification of the rectangle insert. The white star symbol * marks a 15micron vessel and the white arrow points to a 27 microns vessel. Dark lines and numbers represent the line measurements and vessel numbers as performed in ImageJ.
