# Supplementary material for: Experimental and theoretical model of microvascular network remodeling and blood flow redistribution following minimally invasive microvessel laser ablation
Source: Sci Rep. 2024 Apr 16;14:8767. doi: 10.1038/s41598-024-59296-w (PMC11021487; doi:10.1038/s41598-024-59296-w)
Supplement: Supplementary file 2 — Supplementary Information 2. [file 41598_2024_59296_MOESM2_ESM.pdf]

Mouse	Arterial Segments						Venous Segments					
	Proximal		Medial		Distal		Proximal		Medial		Distal	
	Number	Diameter	Number	Diameter	Number	Diameter	Number	Diameter	Number	Diameter	Number	Diameter
A	13	52.1 ± 19	23	44.8 ± 10.1	10	50.6 ± 11.7	23	60 ± 48.6	20	45.3 ± 24.5	13	73.5±38
B	35	34.8 ± 15.5	31	31.2 ± 16.4	18	33.9 ± 14.8	19	80.1 ± 82	81	48.1 ± 55.1	31	43 ±53.1
C	5	26.8 ± 5.8	7	25 ± 9.9	15	29.5 ± 15.4	34	34.3 ± 16	39	43.8 ± 27.8	15	45.3 ±26.9
D	14	39.3 ± 13.7	20	31.4 ± 16.9	8	43 ± 17.2	23	115.1 ±100.7	25	41.7 ± 50.7	12	128.9 ± 66
E	12	30.2 ± 14.3	25	38.2 ± 19.4	18	23.1 ± 7.6	19	75 ± 60.5	27	51.6 ± 46.5	11	19.2 ± 6.8

Supplementary Table 1. Number and average diameter ± standard deviation of arterial and venous vessel segments included in the study before laser ablation for mice A-E shown in sFig.1.

M	A				B				C				D				E			
	D0	D4	D12	D17	D0	D1	D1+	D5	D0	D3	D6	D18	D0	D2	D2+	D4	D0	D6	D13	D20
P	-1.1 ± 37	-24.4 ± 31	-10.6 ± 39	19.4 ± 61	-6.4 ± 46	9 ± 35	-23.8 ± 40	8.4 ± 92	8.5 ± 40	23.4 ± 33.3	56.7 ± 59.3	55.4 ± 70.3	8.1 ± 33.2	17.4 ± 40.6	3.4 ± 4 6.1	15.7 ± 56	-26.4 ± 36	19 ± 61	80 ± 70	40.7 ± 61
M	11 ± 31	-23.4 ± 22	12 ± 24	26 ± 37	4 ± 24	28 ± 42	-6 ± 51	39 ± 74	30 ± 71	74 ± 96	105 ± 120	61 ± 81	18 ± 39	20 ± 50	9 ± 42	25 ± 61	-5 ± 29	64 ± 59	121 ± 88	57 ± 66
D	9 ± 25	-36 ± 18	-7 ± 22	0 ± 29	-4 ± 29	19 ± 45	-35 ± 26	51 ± 44	51 ± 37	66 ± 68	135 ± 72	110 ± 102	11 ± 21	-8 ± 32	-6 ± 17	N/A	14 ± 48	65 ± 63	208 ± 80	121

Supplementary Table 2. The percentage change of artery diameters from intact values for mice A-E shown in sFig. 1. M - mouse. P- Proximal, M- Medial, D-Distal areas away from the closest ablation for each vessel segment. D0, D4, D12, etc. represent the days of observation at ablation (D0) and post-ablation. N/A - the respective arterial vessel segments in the distal area were not available for measurement due to skin movement during the observation period.

M	A				B				C				D				E			
	D0	D4	D12	D17	D0	D1	D1+	D5	D0	D3	D6	D18	D0	D2	D2+	D4	D0	D6	D13	D20
P	-2.9 ± 16.6	-13.5 ± 17	-8.38 ± 18	8.48 ± 21.5	-3.6 ± 9.3	1.2 ± 8.7	-11.3 ± 12.8	-3.1 ± 21.3	0.9 ± 9.8	5.1 ± 7.4	12.5 ± 11.0	12.2 ± 13.3	2.3 ± 12.1	3.7 ± 13.3	-3.3 ± 13.4	2.3 ± 20.1	-15.3 ± 16.5	-0.4 ± 14.5	20.2 ± 11.3	6.8 ± 14.2
M	6.0 ± 14.5	-9.6 ± 10.1	5.5 ± 11.6	12.6 ± 17.3	-0.3 ± 6.1	4.7 ± 8.2	-6.6 ± 11.9	3.7 ± 16.4	2.9 ± 11.6	10.7 ± 17.7	17.3 ± 16.4	9.1 ± 13.9	4.8 ± 13.3	2.2 ± 14.6	0.7 ± 11.2	6.8 ± 17.2	-4.6 ± 9.6	14.5 ± 10.7	30.7 ± 16.4	14.4 ± 14.7
D	3 ± 11.6	-18.4 ± 10.2	-3.7 ± 11.1	0.7 ± 18	-2 ± 6.5	2.5 ± 8.4	-12.5 ± 10	12.4 ± 7.1	11.8 ± 10.6	10.9 ± 14	31.2 ± 9.4	20.4 ± 25.7	4.1 ± 10	-7.2 ± 14.3	-4.5 ± 8	N/A	0.8 ± 7.6	13.6 ± 11.4	42.3 ± 5.2	17.7

Supplementary Table 3. The delta changes of artery diameters from intact values for mice A-E shown in sFig. 1. M - mouse. P- Proximal, M- Medial, D-Distal areas away from the closest ablation for each vessel segment. D0, D4, D12, etc. represent the days of observation at ablation (D0) and post-ablation. N/A - the respective arterial vessel segments in the distal area were not available for measurement due to skin movement during the observation period.

M	A				B				C				D				E			
	D0	D4	D12	D17	D0	D1	D1+	D5	D0	D3	D6	D18	D0	D2	D2+	D4	D0	D6	D13	D20
P	66 ± 56	5 ± 51	40 ± 159	118 ± 233	2 ± 31	34 ± 35	20 ± 50	-18 ± 90	-5 ± 27	43 ± 97	3 ± 53	219 ± 269	18 ± 47	31 ± 76	39 ± 77	26 ± 71	10 ± 37	14 ± 61	133 ± 186	94 ± 196
M	56 ± 55	27 ± 84	-11 ± 34	45 ± 76	-14 ± 39	32 ± 46	43 ± 49	119 ± 255	-3 ± 40	54 ± 83	32 ± 64	169 ± 239	15 ± 37	44 ± 39	63 ± 47	22 ± 52	21 ± 24	36 ± 97	97 ± 112	39 ± 82

D	46 ± 17	46 ± 38	28 ± 38	56 ± 47	-3 ± 35	39 ± 43	58 ± 71	13 ± 176	4 ± 35	85 ± 63	89 ± 100	86 ± 122	-40 ± 22	-80 ± 26	-25 ± 31	N/A	38 ± 33	55 ± 41	152 ± 12	N/A

Supplementary Table 4. The percentage changes of vein diameters from intact values for mice A-E shown in sFig. 1. M - mouse. P- Proximal, M- Medial, D-Distal areas away from the closest ablation for each vessel segment. D0, D4, D12, etc. represent the days of observation at ablation (D0) and post-ablation. N/A - the respective venous vessel segments in the distal area were not available for measurement due to skin movement during the observation period.

M	A				B				C				D				E			
	D0	D4	D12	D17	D0	D1	D1+	D5	D0	D3	D6	D18	D0	D2	D2+	D4	D0	D6	D13	D20
P	23.0 ± 14	-12 ± 30	-12 ± 39.2	25.6 ± 38.4	-0.9 ± 6	17.5 ± 17.4	-12.6 ± 58.7	-52 ± 81.8	-4.1 ± 9.5	8.3 ± 23	-2.5 ± 13.5	60.8 ± 61.8	-4.7 ± 54.3	3.2 ± 51.7	1.3 ± 71.1	-9.9 ± 66.5	-0.5 ± 23.4	-14.5 ± 39.1	56.7 ± 45.8	26 ± 49
M	22.8 ± 23.3	8.6 ± 25.2	-6.9 ± 12.9	15.6 ± 32.8	-3.9 ± 13	7 ± 13.8	7.6 ± 14.7	6 ± 70.3	-2.7 ± 13	18.6 ± 26.8	11.8 ± 25.1	48.6 ± 65	5.7 ± 17.4	11.1 ± 31.2	17.4 ± 32.7	5.9 ± 31	4.6 ± 14	9.2 ± 44.1	41.1 ± 43.3	13.6 ± 32.7
D	32.3 ± 16.2	34.7 ± 28.4	24.5 ± 28.1	39.6 ± 32.8	1.6 ± 9.8	10.8 ± 13.7	8.6 ± 17	-10.8 ± 42.8	-1.9 ± 11.6	32.0 ± 30.1	37.4 ± 41.8	27.6 ± 52.6	-48.5 ± 33.3	-97.8 ± 45.2	-54.5 ± 52.8	N/A	7 ± 5.7	9.6 ± 7.7	29.7 ± 17.5	N/A

Supplementary Table 5. The delta changes of vein diameters from intact values. M- mouse. The P- Proximal, M- Medial, D-Distal. D0, D4, D12, etc. represent the days of observation at ablation (D0) and post-ablation. N/A - the respective venous vessel segments in the distal area were not available for measurement due to skin movement during the observation period.

M	A				B				C				D				E			
	D0	D4	D12	D17	D0	D1	D1+	D5	D0	D3	D6	D18	D0	D2	D2+	D4	D0	D6	D13	D20
Outward/inward remodeling	Y	Y	Y	Y	Y	Y	Y	Y	Y	Y	Y	Y	Y	Y	Y	Y	Y	Y	Y	Y
Collateral Outward Remodeling	N	Y	Y	Y	N	Y	Y	Y	N	Y	Y	Y	N	N	Y	Y	N	Y	Y	Y
Arterial/venous segment reopening	N	Y	Y	Y	N	Y	N	Y	N	N	N	Y	N	Y	N	Y	N	N	N	Y
Permanent Segment Occlusion	Y	Y	Y	Y	Y	Y	Y	Y	Y	Y	Y	Y	Y	Y	Y	Y	Y	Y	Y	Y

Supplementary Table 6. Summary of observed remodeling patterns following laser ablation. Both outward and inward remodeling of arterial and venous segments were consistently observed at all time points, as indicated in Supplementary Tables 2-6. D0, D4, D12, etc. represent the days of observation at ablation (D0) and post-ablation. Collateral outward remodeling and reopening of arterial and venous segments were observed after day 0 post-ablation. Permanent segment occlusion was achieved with each laser ablation. While certain vessels remained occluded throughout the observation period, others reopened over time.
