# Supplementary material for: Experimental and theoretical model of microvascular network remodeling and blood flow redistribution following minimally invasive microvessel laser ablation
Source: Sci Rep. 2024 Apr 16;14:8767. doi: 10.1038/s41598-024-59296-w (PMC11021487; doi:10.1038/s41598-024-59296-w)
Supplement: Supplementary file 14 — Supplementary Information 3. [file 41598_2024_59296_MOESM14_ESM.pdf]

SUPPLEMENTARY VIDEOS

1) Flow oscillations in the artery of Ablation Region 1.

Video 1a.

Intravital microscopy of Region 1 on day 6 after ablation. The brown area marks the region of ablation. The flow in the arterial branch distal from the ablation site has reversed, and now supplies the tissue at upper left of the field of view. Note also the smaller vessels (arrowhead) carrying the return flow to the vein, bypassing the site of ablation of the vein. Toward the end of the video, the microscope is switched to the dsRed channel to show the smooth muscle cells, and then to the GFP channel to show the TIE-2 positive cells.

Video 1b.

Intravital microscopy of Region 1 on day 20 after ablation. The arterial segment is now reperfused from the feeding artery, and flow direction has reversed, achieving the correct direction in this segment (arrow). Downstream, there is still opposing pressure from collateral vessels that compensated for the injury on day 6. This causes stagnation and oscillations in one segment. Note also the significant remodeling of the vein in this region.

Video 1c.

Intravital microscopy of Region 1 on day 23 after ablation. Flow in the reconnected arterial segment is initially sluggish and oscillatory, still affected by the collateral flow coming from downstream (arrowhead). After a few minutes, the flow reverses and there is retrograde flow in this artery. Toward the end of the video, the microscope is switched to the dsRed channel to show the smooth muscle cells.

Video 1d.

Intravital microscopy of Region 1 on day 30 after ablation. The reconnected arterial segment has undergone outward remodeling and has increased in diameter. Flow is now stable and in the correct direction. Toward the end of the video, the microscope is switched to the dsRed channel to show the smooth muscle cells, and then to the GFP channel to show the TIE-2 positive cells.

2) Flow oscillations in the artery in Ablation Region 2.

Video 2a. Intravital microscopy of Region 2 on day 6 after ablation. The downward branch of the ablated artery has maintained patency, although there is a pronounced constriction at the connection. The flow direction in this artery has reversed, driven by higher pressures caused by collateral flow to the left and bottom of the field of view. Toward the end of the video, the microscope is switched to the dsRed channel to show the smooth muscle cells, and then to the GFP channel to show the TIE-2 positive cells.

Video 2b. Intravital microscopy of Region 2 on day 23 after ablation. The connection between the ablated artery and the downward branch is now larger. Note that some flow is coming from the ablated regions of the vessel into small capillaries in the tissue from opposite sides (arrows). Note also that the angle of the junction has decreased compared with day 6 due to remodeling (arrowhead). Toward the end of the video, the microscope is switched to the dsRed channel to show the smooth muscle cells.

Video 2c. Intravital microscopy of Region 2 on day 30 after ablation. The flow in the branch formed by the ablated artery is still unstable (yellow arrowhead). Initially during the observation, flow is retrograde, but it then reverses after a few minutes. Note that considerable flow is entering the smaller branch extending to the left (blue arrowhead). Note also the flow entering the area of ablation through capillaries from the left-hand side and to the left from the right-hand

side (arrows). Toward the end of the video, the microscope is switched to the dsRed channel to show the smooth muscle cells, and then to the GFP channel to show the TIE-2 positive cells.

3) Flow oscillations and remodeling in Ablation Region 3.

Video 3a. Intravital microscopy of Region 3 on day 6 after ablation. The brown areas mark the regions of ablation. Flow in the large vein has rerouted through a series of smaller vessels, which are undergoing outward remodeling to accommodate the flow. Flow in the upper and lower artery segments has reversed (yellow arrowhead), and there is stagnation in the upper artery, distal from the ablation site (blue arrowhead). Toward the end of the video, the microscope is switched to the dsRed channel to show the smooth muscle cells, and then to the GFP channel to show the TIE-2 positive cells.

Video 3b. Intravital microscopy of Region 3 on day 20 after ablation. The vein has further remodeled, achieving a relatively constant diameter along its length. Flow in the upper and lower artery segments is still reversed (arrowheads). Flow in the upper artery is now visible and is also retrograde.

Video 3c. Intravital microscopy of Region 3 on day 23 after ablation. A number of capillaries are visible in the region of ablation, but they do not appear to connect the ends of the ablated segment (arrows). Flow in the upper and lower arteries is still reversed (arrowheads). Toward the end of the video, the microscope is switched to the dsRed channel to show the smooth muscle cells, and then to the GFP channel to show the TIE-2 positive cells.

Video 3d. Intravital microscopy of Region 3 on day 30 after ablation. The vein has undergone further remodeling to become less tortuous. Flow in the upper and lower arteries is still

reversed. The flow from the ends of the ablated artery into the capillary bed is more prominent (arrows). Toward the end of the video, the microscope is switched to the dsRed channel to show the smooth muscle cells, and then to the GFP channel to show the TIE-2 positive cells.
